# Use of Social Information in Seabirds: Compass Rafts Indicate the Heading of Food Patches

**DOI:** 10.1371/journal.pone.0009928

**Published:** 2010-03-29

**Authors:** Henri Weimerskirch, Sophie Bertrand, Jaime Silva, Jose Carlos Marques, Elisa Goya

**Affiliations:** 1 Centre d'Etudes Biologiques de Chizé, Centre National de la Recherche Scientifique, Villiers en Bois, France; 2 Centre de Recherche Halieuthique, Institut de Recherche pour le Développement, Sète, France; 3 Direccion de Recursos Pelagicos, Instituto del Mar del Perú, Callao, Peru; Smithsonian's National Zoological Park, United States of America

## Abstract

Ward and Zahavi suggested in 1973 that colonies could serve as information centres, through a transfer of information on the location of food resources between unrelated individuals (Information Centre Hypothesis). Using GPS tracking and observations on group movements, we studied the search strategy and information transfer in two of the most colonial seabirds, Guanay cormorants (*Phalacrocorax bougainvillii*) and Peruvian boobies (*Sula variegata*). Both species breed together and feed on the same prey. They do return to the same feeding zone from one trip to the next indicating high unpredictability in the location of food resources. We found that the Guanay cormorants use social information to select their bearing when departing the colony. They form a raft at the sea surface whose position is continuously adjusted to the bearing of the largest returning columns of cormorants. As such, the raft serves as a compass signal that gives an indication on the location of the food patches. Conversely, Peruvian boobies rely mainly on personal information based on memory to take heading at departure. They search for food patches solitarily or in small groups through network foraging by detecting the white plumage of congeners visible at long distance. Our results show that information transfer does occur and we propose a new mechanism of information transfer based on the use of rafts off colonies. The use of rafts for information transfer may be common in central place foraging colonial seabirds that exploit short lasting and/or unpredictably distributed food patches. Over the past decades Guanay cormorants have declined ten times whereas Peruvian boobies have remained relatively stable. We suggest that the decline of the cormorants could be related to reduced social information opportunities and that social behaviour and search strategies have the potential to play an important role in the population dynamics of colonial animals.

## Introduction

Animals living or gathering in groups at certain period of their life can use personal information obtained from environmental cues and social information from the behaviour of congeners to make decisions [1], [2]. The balance between personal and social information in decision making use is likely to reflect the optimal adjustment to exploit the most reliable information. Today evidence is accumulating that the use of social information is common in nature [3], [4] that the larger groups should favour the use of social information and improve the ability to make correct decisions compared with smaller groups[5].

The sight of hundred of thousands or millions of seabirds gathering together into a single colony has always fascinated observers, and stimulated questions about the interest of so many individuals concentrating at the same place. Apart from rare cases where constraints on accessibility to nest sites or food resources occur, colonies are considered as an efficient strategy to limit predation [6], but there are many related costs (diseases, ectoparasite infection, competition for food or nesting sites [7]. Before the concept of social information was developed [4], Ward and Zahavi [8] suggested that colonies may serve as a site of information exchange about the location of food, also known as the information centre hypothesis (ICH). Because sea birds rely on food resources that are patchily distributed, with location being highly variable in space and time [6], [9], breeding in colonies could provide the opportunity for individuals to obtain information about the location of favourable food patches by watching the behaviour of other individuals when returning from, or leaving for feeding grounds. The empirical studies that have tested this hypothesis came to mixed conclusions (see review in Richner and Heeb (1995) [10]). However, most empirical studies have focused on the demonstration that individuals follow from the colony successful foragers heading to feeding grounds, whereas information exchange may occur outside the colony and be based on the observation of the returning successful individuals [11], [12].

Several alternative hypotheses, related to the ICH, have been proposed to explain the advantage of breeding in large groups, based on purely individual selection. The local enhancement hypothesis [13], [14] suggests that the increase in density of birds foraging from a colony improves the probability of discovering unpredictably distributed food patches. The recruitment centre hypothesis [10], [15] predicts that communal feeding is important for successful foraging at feeding patches, therefore birds recruits congeners at colonies. Otherwise individuals may simply use personal information based on past foraging experience and memory [16] to return to foraging grounds. This strategy is relatively common when seabirds search for resources spatially aggregated in a predictable way [9].

More recent modelling studies have suggested that the different hypotheses related to ICH are probably not exclusive, and should be considered in a common framework [17], and that seabirds may use a mixture of searching strategies [18]. Therefore they may use social information as well as personal information so as to maximise food brought to the offspring and minimise time spent commuting and searching [19], [20]. However, there is a need for more empirical studies, in particular those based on the study of individual behaviour from the colony to food patches. These studies are difficult to undertake in natural conditions, but with the advances in telemetry miniaturisation have opened up new methods to empirically address these questions [21].

Guanay cormorants and Peruvian boobies are the main guano producers breeding along the Peruvian coast. They concentrate in huge colonies that historically could group hundreds of thousands of individuals in a single colony, representing one of the world's most spectacular aggregations of seabirds [22]. They rely mainly on Peruvian anchovy ([23], [24] that has sustained, until recently, the world largest single-species fishery [25]. The black plumaged cormorants, nest in extremely dense colonies and are social foragers forming endless columns moving from colonies to feeding grounds whereas the white plumaged boobies breed in large, but less dense colonies [22], [26]. Over the past 50 years, Guanay cormorants have dramatically decreased from c.21 millions birds to 2 millions, whereas Peruvian boobies have remained relatively stable at 2 millions birds [27]. Reasons for the decline of Guanay cormorants have been related to successive El Niño events and competition with the industrial fishery which developed in the 1950s [28]. The observation that the populations of cormorants and boobies show different trends although the two species breed together and feed on the same prey led us to hypothesise that differences in foraging ecology between species may be implied in the differential trends of the species populations. In particular, the extent of personal versus social information used to find food patches may differ between species.

The aim of the study is to examine in these extremely colonial seabirds whether there is evidence of an information transfer between conspecifics individuals about the location of food resources on or in the vicinity of the colony and how it may be conveyed to congeners. We also examine whether we could find indication of information transfer between species. Because of the differences between the two species in plumage characteristics and aggregative behaviour while foraging, we hypothesise that the extent of use of personal versus social information may differ between the two species. We combined a study of individual tracking using high precision miniaturised GPS and Time Depth Recorders and colony based observations on the movements and behaviour of groups and their outward and return flight directions.

## Materials and Methods

### Study site and field methods

The study was carried out between 22 November and 10 December 2008 on Isla Pescadores (11.775°S, 77.265°W), a small island located 7.5 km off the central coast of Peru. During the study period an estimated 190,000 Guanay cormorants and 15,000 Peruvian boobies were breeding, mainly rearing small to large chicks. First we made observations of groups leaving and returning to the colonies from a vantage point located on the summit and centre of the island (altitude 110 m) where a 360° view of the sea was possible. Every hour from dawn to dusk, using 10×32 binoculars and electronic compass, the same observer (HW) recorded the inward and outward flight directions with respect to central submit of the island of every group (>10 individuals) of the two species within a 2 km range from the island, as well as the size of groups. We considered only commuting groups flying just over the sea up to 20–30 m above sea level and not the groups circling the island high in the sky. After a few days of observation we discovered that Guanay cormorants form rafts, i.e. cluster of individual on the sea surface, when departing. The bearing of the rafts where birds concentrate after leaving the colony and before heading for a foraging trip was noted according to the centre of the island.

Second, we equipped with 51 Peruvian boobies (average mass 1520 g) with Gipsy GPS (25–30 g, Technosmart, Italy) and 20 Guanay cormorants (2150 g) with MiniGPSlog (30 g, Earth and Ocean GPS, Germany). Birds attending their chick on the nest were selected randomly in the colony and captured using a fishing rod equipped with a noose. The GPS recorded locations at 1 sec or at 30 sec intervals and were attached with Tesa tape on the tail feathers (boobies) or on the back feathers (cormorants) for 1 to 9 successive trips, giving a total of 165 and 46 foraging trips for boobies and cormorants respectively. All birds were recaptured except one cormorant but we were not able to retrieve data from two GPS deployed on cormorant and 3 on boobies. In addition all cormorants and 15 boobies fitted with GPS receivers were also equipped with Time Depth Recorders (TDR) recording at 1 sec intervals (G5 (3 g), CEFAS Technology, UK) fixed on the leg with a metal band.

Daily weather conditions were very similar through the study period, with no wind and foggy conditions in the morning, clearing at midday with a south easterly wind increasing to moderate and decreasing in the evening, and could not account for the continuous changes in flight direction and raft position.

### Data analysis

Data extracted from TDR and GPS were merged into a single file that was analysed to calculate the basic foraging parameters such as distance covered and speed between locations, time spent foraging, maximum foraging range and total distance covered. We also calculated from GPS data the bearing at departure from the colony and when returning and the zone of feeding (where birds use Area Restricted Search behaviour [29]–i.e. increase sinuosity - and/or dive, take off and land actively [30]). Because some individuals were tracked for multiple successive trips, we analysed foraging parameters (maximum range, time spent foraging) using mixed-model analyses of variance (ANOVAs; module VEPAC in STATISTICA 8) to overcome issues of pseudoreplication. Foraging parameters were considered as dependent variables, species were added to the model as fixed factors and bird identity was included as a random factor. Frequencies of occurrence of trip classes were compared between species using Chi-square tests. We examined circular correlations between bearings to test whether individual birds use a memory based strategy whereby they keep the same bearings at departure during two successive trip (suggesting a persistence of decisions to take a bearing [31]), or same bearings at departure than that taken when returning during the previous trip (suggesting predictability[9]). Circular correlations were also conducted (a) on tracking data on the individual angles between bearings of consecutive departures or between bearings at return and at next departure, and (b) on observations of groups and columns on the angles of bearing at departure and return of columns, and on the position of the raft. All circular statistical analyses were performed using the Package *circular* version 0.3–8 (Correlation Coefficient for Angular Variables, Watson two samples Test of Uniformity) in R.

## Results

**Figure 1:**
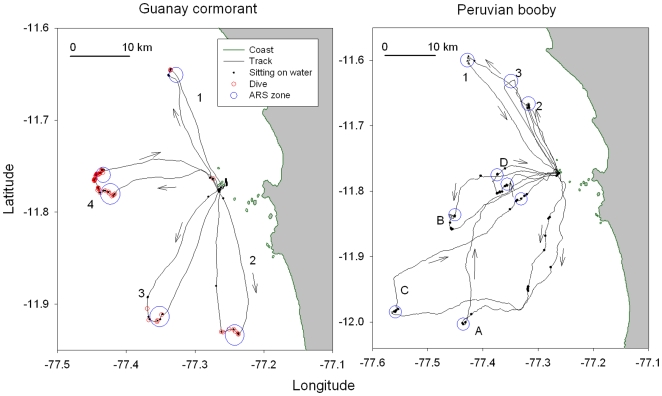
Foraging trips of Guanay cormorants (left) and Peruvian boobies (right) tracked with GPS. Left: four successive return trips of a Guanay cormorant (1–4). Right: three successive return trips of a Peruvian booby (1–3) and four successive tracks (A–D) of a second individual Peruvian booby (looping course A and C, return trip B and D). Arrows indicate the flight direction; dots indicate sitting on the water, small red circles the deep diving events, blue circles zones of area restricted search (ARS).

When rearing chicks guano birds are typical central place foragers, making foraging trips from the nest to search for food at sea. When one parent forages at sea, the partner guards the chicks until it is relieved by the returning member of the pair. Both species foraged strictly during the day time. Individual tracking shows that both boobies and cormorants use two distinctive movements. They both use ‘Return Trips’, leaving the colony to a particular bearing that is kept until they reach a feeding zone and then return straight to the colony, making the outward and return routes parallel, with an angle <10° (Fig. 1). They also both use ‘Looping Trips’ whereby birds change direction several times before feeding (or not) and then return to the colony from a bearing different from that taken during the outward phase, with an angle >10° (Fig. 1). Feeding zones are clearly visible, as indicated by circling over a particular restricted area, diving actively, sitting on the water and taking off in successive bouts (Fig. 1). Feeding zones were found in all Return trips except one and in 81.19% of Looping trips (χ^2^_1_ = 5.3, P = 0.0211) indicating that in most trips birds have encountered a prey patch. Furthermore, all birds captured just after returning from the sea regurgitated fishes (anchovies mainly) confirming that most birds return only after a successful fishing. Return Trips represent 62% of trips for Guanay cormorants and only 39.8% for boobies (χ^2^_1_ = 5.8, P = 0.0163). The duration of foraging trips was longer for cormorants than for boobies (Mixed ANOVA, 2.0±0.8 h versus.1.2±0.5 h F_1,39_ = 32.9, P<0.001) and was longer for Looping Trips than for Return Trips (2.3±0.8 h versus 1.7±0.6 h for cormorants and 1.4±0.6 h versus 0.9±0.3 h for boobies). The maximum range was similar for the two species (20.2±11.5 km for boobies versus 18.9±6.1 for cormorants, F_1,39_ = 0.5, P = 0.480), but Looping Trips had longer range than Return Trips (21.1±9.7 versus 16.6±5.8, F_1,39_ = 10.4, P = 0.004). In both species, feeding zones were never at the same location from one trip to the next except in one case for each species, with long distance between successive feeding zones (distance between successive feeding zones = 19.9±11 km for cormorants and 16.9±12.2 for boobies F_1,29_ = 0.5, P = 0.461). There was no difference whether we consider distance between successive foraging zones during the same day, or from one day to the next.

**Figure 2:**
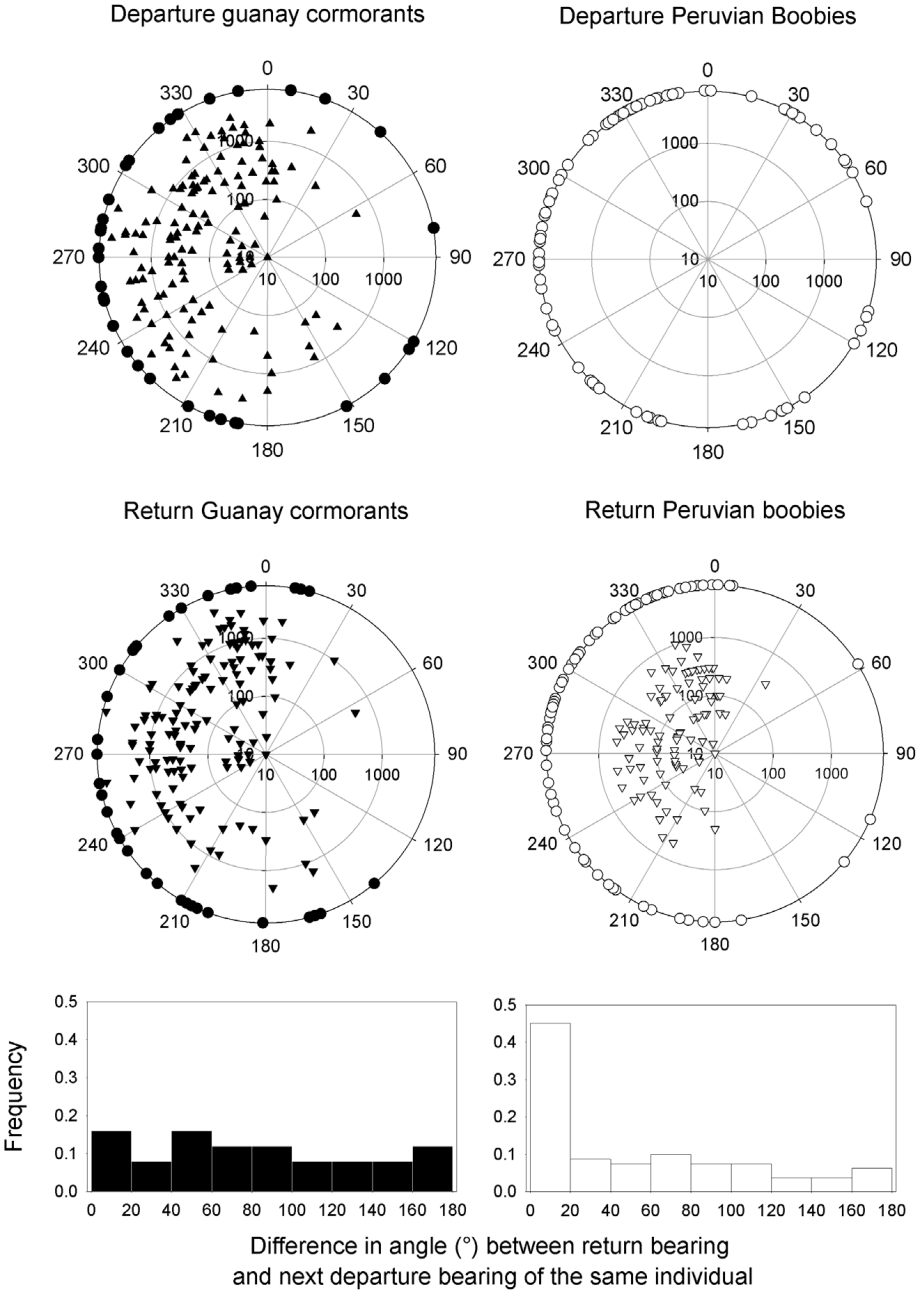
Bearings taken by groups according to the centre of the island and difference in angle between the return bearing and the departure bearing of Guanay cormorants and Peruvian boobies. Upper four figures: Bearing (in°) taken by groups according to the centre of the island and size of groups (from 10 to 10,000) of Guanay cormorants (black) and Peruvian boobies (white) leaving the island (▴) and returning to the island (▾). The circles indicate the bearings at departure and when returning taken by individuals tracked with GPS. Lower two figures: difference in angle between the return bearing and the departure bearing during the next foraging trip of individuals tracked with GPS.

The bearings taken by individual tracked birds departing from the colony or returning from the feeding zones or of groups of birds observed were mainly directed toward north, west and south, with few trips heading to the east (Fig. 2), and do not differ between species (Watson two sample test, U = 0.922, P = 0.187). Individual birds tend to change direction from one trip to the next (Fig. 1). For GPS tracked Guanay cormorants, there is no correlation between the bearing when returning to the island and the bearing of the next outward trip (Table 1) confirming that birds do not return to the same feeding zone from one trip to the next. For boobies there is a tendency for birds to take the same direction than that of the return part of the previous trip, only when successive trips were carried out the same day (Table 1).

**Table 1:** Circular correlation coefficients between angles for Guanay cormorants and Peruvian boobies fitted with GPS.

	Foraging flight bearings	Guanay cormorant			Peruvian boobies		
Test for:							
		Coeff	Stat	P	Coeff	Stat	P
Parallelism	Departure trip 1 vs Return trip 1	0.90	4.8	<0.001	0.781	6.6	<0.001
Persistence	Departure trip 1 vs Departure trip 2	0.178	0.9	0.362	0.02	0.2	0.862
Predictability (all days combined)	Return trip 1 vs Next Departure trip 2	0.202	1.0	0.311	0.189	1.4	0.147
Predictability (same day only)	Return trip 1 vs Next Departure trip 2	0.296	1.1	0.265	0.305	2	0.052

**Figure 3:**
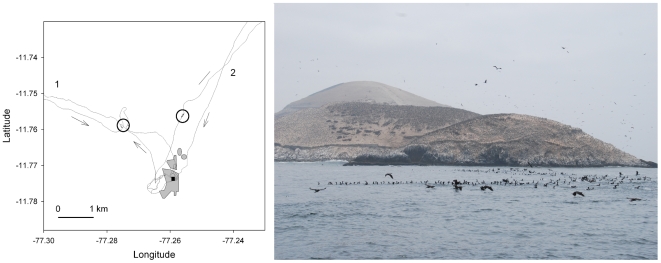
Movements of a Guanay cormorant in the vicinity of the colony and view from the sea of a compass raft. (Left) Fine scale movements of a Guanay cormorant tracked by GPS at 1 sec interval in the vicinity of Isla Pescadores (in grey). Two successive foraging trips (1 and 2) from the nest (black circle). The location of the compass raft visited after departure from the colony is indicated by a circle (circle) and the arrows indicate flight direction. (Right) Photograph taken from the sea of a compass raft, with the colonies of seabirds on Isla Pescadores in the back ground.

**Figure 4:**
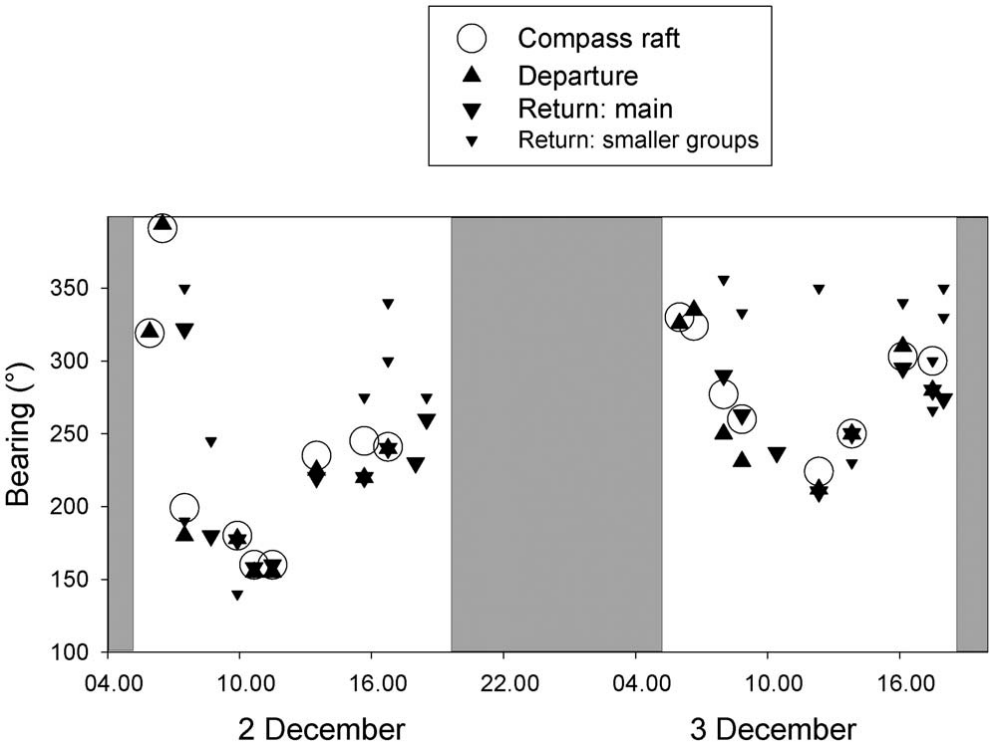
Changes in the bearings according to the centre of the island of the compass raft (circle) and of the departing (▴) and returning (▾) groups of Guanay cormorants during two consecutive days. In some cases, when opposing arrows overlay, they appear as a star.

**Figure 5:**
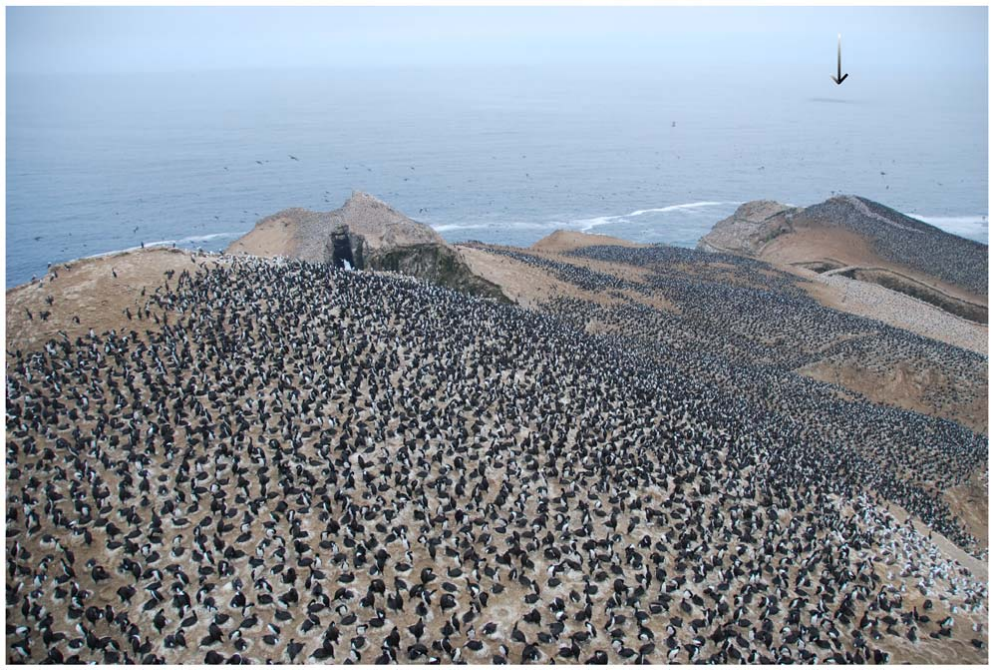
View from the summit of the island showing part of a large aggregation of nesting guano birds on Isla Pescadores, with compass raft at sea. The photograph shows part of the seabird aggregations dominated by Guanay cormorants (black plumage), with Peruvian boobies (white plumage) on the edge of the mains groups of cormorants.The black arrow indicates the location of the compass raft at sea.

Individual tracking of Guanay cormorants shows that after departing the colony, birds circle the island and then land on water 300 m–2 km off the island for a few seconds before heading for a particular direction (Fig. 3a). This stereotyped behaviour has been observed in all tracked birds. Observations from the vantage point confirm that birds circled around the island in small groups, and then joined the raft. At any time this raft grouped an average 305±163 individuals on the water (range 50–1000). Remarkably there was only a single raft around the island at once, but over time its position continuously changed (Fig. 4). The raft formed one-two hour after sunrise, remained present through the day and started vanishing 1–2 hours before sunset when no more birds were leaving (Fig. 4). Since birds are continuously landing, shaking wings and taking off, the raft can be seen from a long distance from the colony as a point of foaming water (Figs. 3, 5). Individual birds only stayed in the raft for 5–30 seconds and then took off in large groups, queuing one after the other, all in the same direction, forming long columns of successive groups, regularly uninterrupted up to the horizon. Interestingly, departing groups in columns flew just above the sea surface, whereas returning groups in columns flew at an altitude of 10–30 m above sea surface. The raft was aligned with the columns of birds returning from the sea, specifically with the largest returning groups (Circular Correlation coefficient, r = 0.610, F = 4.6, P<0.001, Fig. 3). Rafting off the colony at departure does not occur in Peruvian boobies: they departed solitarily or in small groups of a few birds, and returned alone or in small to medium sized groups (Fig. 2), often included in the large formations of Guanay cormorants. The bearings of the largest groups of cormorants and boobies were strongly correlated (Circular Correlation, r = 0.981, F = 6.6, P<0.001).

## Discussion

The first, and most important, result of this study indicates clearly that Information Transfer does occur in a colonial seabird through a specific signalling behaviour, by forming a compass raft. The function of this raft appears to be a signal that is well visible from the colony or from birds that are circling in flight the island (Fig. 3b), pointing the heading to be taken to birds leaving the colony. By using this compass raft, Guanay cormorants rely entirely on the information obtained from returning congeners. Therefore the raft plays the role of a signal to other congeners [15]. It is remarkable to note that any Guanay cormorant leaving the colony joint the compass raft before heading for feeding grounds. Just before landing and from the raft they are probably able to detect the bearing of the incoming columns that stay high in the sky. Thus Guanay cormorant use social information made available from the largest columns returning from a distant prey patch. This is possible only because the majority of the returning birds have been successful in a prey patch that they have reached either directly by a Return Trip, or after a longer time searching through a Looping Trip. But as soon as they have had found a food patch, they returned in a straight line to the colony making the heading of the returning columns as reliable social information. The social information is transferred through the compass raft and updated continuously through the alignment of the compass raft to the returning largest columns (i.e. probably the most successful groups).

After attending their chicks at the colony, birds are relieved by their partner and return at sea, by joining first the compass raft. Therefore they are able to use the most recent information available from congeners returning from a foraging patch. By comparison if they had to rely on their own memory of their previous trip, the information would be less updated, because of the time spent attending the chick. This matches the theory of information centre that predicts that the duration of a food patch should allow at least one return trip [8]. For Guanay cormorants, the basic conditions required for a colony to operate as an information centre are fulfilled, i.e. food patches are ephemeral but last at least several hours. Colony members can easily detect successful foragers, not directly from the colony but from the nearby compass raft [7], [8], [10]. This foraging strategy has probably been selected for the exploitation of food resources whose distribution and availability according to the central place colony may change rapidly over time. These characteristics apply to the main prey of guano birds, the Peruvian anchovy, an extremely abundant epipelagic fish, patchily distributed in space and in time [32]. The location of patches available for guano birds probably changed continuously as suggested by the continuous change in flight direction from one trip to the next in the case of Guanay cormorants, but it also applies to boobies. When birds following columns arrive on a prey patch that has been depleted, they disperse from there and search for another prey patch and this probably results in Looping Trips. Birds return to the colony from this new patch, i.e. from a different bearing, that is detected by the compass raft which then changes direction to adjust to the new bearing of incoming columns.

The behaviour of clustering before heading to feeding grounds acts as a recruitment display not unlike to the complex signalling performed by Ravens to attract congeners [33]. In seabirds, the signalling through rafting could have evolved from the necessity for birds to drink and wash their plumage after a shift at the colony incubating or brooding chicks. In Guanay cormorants this function is rather marginal since a minority of the birds joining the compass raft actually either washes or drink before taking off. The use of rafts for information transfer probably occurs in other seabird species. Indeed many species of colonial seabirds such as albatrosses, alcids or gannets form rafts off the colonies before heading for the sea, but the transfer of information through rafts has been overlooked so far. In Murres Burger [12] suggested that information transfer might not be passed in the colony itself. He noted that 69% of the outcoming birds splashdown from the colony before heading to the sea and he suggested that birds sitting on the ocean in the vicinity of the colony might be well positioned to gain information on the location of prey patches [12], [34]. Observations on albatrosses also indicate that before departing for the sea, birds group in a compass raft off the colony before taking off in small groups, often following a leading individual (H.W. pers. observation). Searching strategies may also be different within the same species according to the site. For example in murres, compass rafts are probably used when patches are unpredictable [12] but memory and local enhancement are used when prey location is predictable and reliable over longer periods of time [18]. Theoretically the coexistence of different search strategies in the same species should yield to a stable strategy [17].

A second important result of this study shows that two highly colonial species, breeding on the same site and feeding on the same prey, can have different searching strategies. When leaving the colony, individuals of each species base their decision to take a particular bearing by using different cues, either by watching congeners and the compass raft, or by using memorised personal observations. The existence of two different search strategies is probably related to the way each species exploit prey patches. Guanay cormorants have a dark plumage that make them cryptic at distance and form columns to reach feeding grounds. They use social fishing that requires foraging in immense groups for successful hunting. They dive in large numbers under fish shoals at depths 10–50 m (H.W. unpublished [35]) to drive fish to the surface [26]. Thus they rely mainly on social information and use colonies as recruitment centre, recruiting congeners through the compass raft. We did not find evidence of information transfer between conspecifics in boobies which did not form rafts off the colonies. Peruvian boobies have a white plumage, conspicuous at a long distance when in flight or plunging, which probably favours local enhancement [36], [37]. They leave colonies solitarily or in small groups and rely mainly on personal information when making a decision about heading. They only feed on prey available close to the surface (average 2 m, maximum 6 m H.W. unpublished data) and can hunt solitarily (pers. obs, [26]). While offshore they can congregate in large numbers through the recruitment of other individuals that probably use network foraging [7], [37]. The observation that Peruvian boobies tend to head toward the direction taken when they returned to the colony several hours earlier, suggests the use of personal information such as a memory based search strategy [31]. However, they almost never return to the same feeding sites from one trip to the next, and could take this heading and search a neighbouring patch because the original one is depleted or no longer available.

We found no evidence that information on direction bearings of food patches may be obtained from other species at or around the colonies. Since boobies leave the colony solitarily it was not possible to test whether they use the compass raft or the columns of cormorants, but our results show that boobies return from the same direction than cormorant, often in small groups included in the large columns of cormorants. However offshore there are several pieces of evidence that suggest that some species such as boobies join feeding groups of cormorants and that plunging boobies constitute an attractive signal for several seabird species [26].

Historically Guanay cormorants used to be ten times more abundant than Peruvian boobies but nowadays both species have similar population size, with 2 million birds of each species [27]. In the early twentieth century several authors were impressed by the unbroken columns of Guanay cormorants heading from the colony to the feeding grounds [22]. The reduction of anchovies stocks in the 1970s has probably led to the crash of the cormorant population at this time [27]. it has continued to decline ever since, whereas Peruvian boobies, who feed on the same prey, have remained stable. The different trends suggest a deterioration of conditions other than food availability for cormorants but not for Peruvian boobies, after the 1970s crash. The different search strategies and reliance on personal versus social information may be involved in the different trends observed. Indeed, if environmental conditions change, a search strategy may become less optimal and result in population decline. The search strategy of Guanay cormorants relies on social information, the use of compass rafts aligned to unbroken columns returning from a feeding ground and on large prey patches. This strategy is probably less optimal if columns are no longer continuous and prey patches less abundant and short lasting. Therefore smaller size populations make use of social information and decision-making less efficient [5]. Conversely Peruvian boobies could be less susceptible to changes in abundance of prey because of their solitarily searching behaviour. These results underline the potential importance of social information for the evolution of life-histories [1], [38]) and for predicting the response of populations to environmental variability [39]. This is also important to consider in the context of longer term evolutionary changes since over the last 10 centuries anchovies, and probably their predators, were much less abundant than they are nowadays [40] suggesting the need for a rapid adaptation to the respective use of personal versus social information.
